# Improved Insulation Properties of Polypropylenes in HVDC Cables Using Aqueous Suspension Grafting

**DOI:** 10.3390/ma15186298

**Published:** 2022-09-10

**Authors:** Yiyi Zhang, Keshuo Shi, Chunyan Zang, Wenchang Wei, Chuanhui Xu, Junwei Zha

**Affiliations:** 1 Guangxi Key Laboratory of Intelligent Control and Maintenance of Power Equipment, Guangxi University, Nanning 530004, China; 2 College of Electical and Electronics Engineering, Huazhong University of Science and Technology, Wuhan 430074, China; 3 School of Chemistry and Chemical Engineering, Guangxi University, Nanning 530004, China; 4 School of Chemistry and Biological Engineering, University of Science and Technology Beijing, Beijing 100083, China

**Keywords:** aqueous suspension grafting, polypropylene, HVDC cable insulation, DC breakdown strength, space-charge suppression, 4-methylphenylene

## Abstract

Owing to its lack of crosslinking, polypropylene (PP) is considered an environmentally friendly alternative to crosslinked polyethylene as high-voltage direct current (HVDC) cable insulation. However, pure PP can accumulate space charges under a HVDC, and thus must be modified for use as an insulating material for HVDC cables. In this study, 4-methylstyrene is grafted onto PP using an aqueous suspension grafting method to improve its properties. The effects of the swelling time, reaction time, and 4-methylphenylene concentration on the reaction were investigated. The optimum process conditions were determined, including an optimum grafting ratio of 0.97%. The volume resistivity, ability to suppress space-charge accumulation, and DC breakdown strength of modified PP were also studied. Modified PP with a grafting ratio of 0.88% showed optimal space-charge suppression and the highest volume resistivity and breakdown strength. The work will facilitate the design and development of more efficient insulation materials for HVDC cables.

## 1. Introduction

High-voltage direct current (HVDC) cables are widely used in submarine and island power transmission, among other applications. The voltage rating and operational reliability of such power cables are determined by their level of insulation. It is imperative to further develop HVDC cable insulation materials for various high-voltage applications to contribute to the development of DC transmission technologies [1].

Crosslinked polyethylene (XLPE) is widely used as an insulating material for HVDC cables, owing to its excellent electrical and mechanical properties [2,3]. However, XLPE is difficult to recycle and persists in the environment, owing to its extremely slow degradation, necessitating the development of environmentally friendly cable insulation materials. Polypropylene exhibits excellent electrical insulation and high resistance to chemical corrosion, along with a high tensile strength, ratio strength, and elastic modulus. Polypropylene can also be easily recycled, making it a potential substitute for XLPE [4,5,6,7,8,9].

High-voltage electric fields cause space charges to accumulate in the insulating layers of the cables, leading to electric field distortion and, in turn, insulation breakdown. Methods to suppress the accumulation of space charges in HVDC cables have, therefore, attracted significant attention in recent years [10,11,12,13,14,15,16]. Among these methods, doping of pristine insulating materials with nanoparticles forms numerous deep traps that hinder the movement of charge carriers, thereby inhibiting the accumulation of space charges [17,18,19,20,21,22]. Nanoparticles are typically incompatible with polymers, owing to their high surface activity, which facilitates the agglomeration of nanoparticles inside the polymer, thereby generating defects; this results in the accumulation of high numbers of space charges inside the nanocomposite, which degrades the electrical properties of the composite [23]. These problems can be avoided by grafting some functional groups onto these polymers. PP can be modified with maleic anhydride groups [14], 4-propoxyenyl-2-hydroxybenzophenone [15], by the melt grafting method. The insulating properties of the modified PP are superior to those of pure PP. Grafting can regulate the macroscopic properties of the polymer through molecular-level chemical modification, while avoiding poor dispersion. Grafting functional groups onto pristine insulating materials is, therefore, considered a viable strategy to inhibit the accumulation of space charges. However, despite its short reaction time and strong grafting effect, the high reaction temperature required by the melt grafting method causes significant side reactions that degrade PP. In contrast, the aqueous suspension grafting method is simple, cost effective, environmentally friendly, and proceeds under mild reaction conditions. Furthermore, the PP degradation and graft adhesion processes observed using the melt grafting method are avoided [24,25,26,27]. The aqueous suspension grafting method has been used to synthesize PP-g-styrene (PP-g-St) graft copolymers, wherein St is grafted onto the main chain of PP macromolecules, and the grafting reaction occurs primarily in the amorphous region of PP [28].

Building on this previous work, in this study, 4-methylstyrene was grafted onto PP using the aqueous suspension grafting method. The grafting effect was characterized by infrared spectroscopy, and the optimal mass ratio of the reactants was evaluated. The volume resistivities, DC breakdown field strength, and space-charge suppression capability of the resulting modified PP-based materials were analyzed. This paper presents a novel, environmentally friendly method for the fabrication of insulation materials for HVDC cables.

## 2. Materials and Methods

### 2.1. Materials and Instruments

Industrial-grade PP powder (T30S, Sinopec Daqing Petrochemical Co., Ltd., Beijing, China) was dried and used as isotactic PP; the particle size is 30 μm and the physicochemical parameters of T30S are as follows: the density is 0.9 g/cm^3^, the melt flow index is 3.3 g/10 min, the isotactic index is 95.0–99.0, granular ash is ≤0.03, the tensile yield stress ≥27.0. Xylene, 4-methylstyrene, and benzoyl peroxide were provided by Shanghai Aladdin Biochemical Technology Co., Ltd., Shanghai, China, and acetone was acquired from Tianjin Fuyu Fine Chemical Co., Ltd., Tianjin, China.

A flat vulcanizer (Guangdong Lina Industrial Co., Ltd., Dongguan, China) was used, along with a digital display intelligent temperature control magnetic stirrer (GongyiYuhua Instrument Co., Ltd., Gongyi, China). Infrared spectroscopy was performed using a Nicolet™ iS50 FTIR spectrometer (Thermo Fisher, Waltham, MA, USA). The reaction temperatures were measured using a synchronous thermal analyzer (DSC; NETZSCH Instruments GmbH, Germany). The resistivities and impedances of the prepared samples were measured using a Keithley 6517B electrostatic tester and a Concept 80 broadband dielectric tester (Novocontrol GmbH, Montabaur, Germany), respectively. The dielectric breakdown strength of the samples was measured using a breakdown tester (Beijing Huace Testing Instrument Co., Ltd., Beijing, China). Finally, the space-charge distribution in the samples was analyzed using a space-charge distribution tester.

### 2.2. Materials Preparation

**Figure 1:**
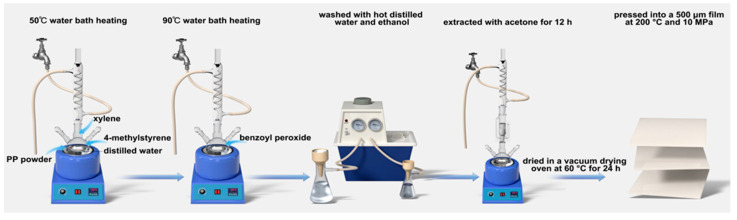
Grafting reaction steps.

**Figure 2:**
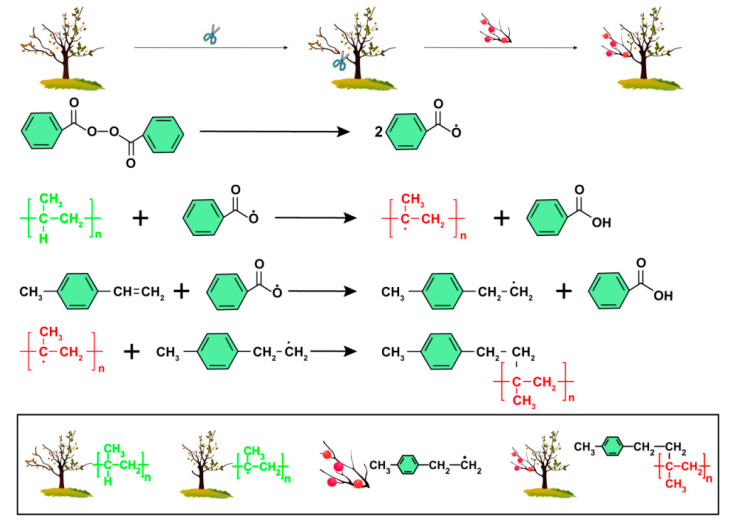
Grafting reaction mechanism.

The PP powder, 4-methylstyrene, xylene, and distilled water were added to a three-necked flask with a condenser. This reaction mixture was heated to 50 °C in a water bath, stirred, and left to swell. After the swelling had progressed for a specified time, the flask was removed from the bath. The flask was again heated at 90 °C, before benzoyl peroxide was added and continuously stirred for the reaction. The process was stopped after the reaction progressed for a specified time. The product was subsequently washed with hot distilled water and ethanol, filtered to remove other impurities, and extracted with acetone for 12 h. The extracted product was then dried in a vacuum drying oven at 60 °C for 24 h, and the final drying was performed using a flat vulcanizer. The product was pressed into a 500 μm film at 200 °C and 10 MPa. The grafting reaction steps and mechanisms are shown in Figure 1 and Figure 2. As shown in picture 2, under the action of the initiator benzoyl peroxide, the double bond on the graft group 4-methylstyrene is opened, and undergoes a radical polymerization reaction with polypropylene to form a rafted product.

### 2.3. Material Property Testing

The Fourier transform infrared spectra of pure PP, modified PP, and 4-methylstyrene were recorded in 32 scans at 3000–500 cm^−1^, using an infrared spectrometer with a resolution of 2 cm^−1^.

The grafting ratio of the grafted material was determined from the ratio of the height of the absorption peak of the C=C bond of the benzene ring to that of the C–CH_3_ absorption peak of PP in the absorbance image, which was obtained from the infrared spectral transmittance map. The relative grafting ratio is calculated by Equation (1).

*Ra*% = *I*_1504_/*I*_1375_ × 100 (1)

where *Ra* is the relative grafting ratio, *I*_1504_ is the height of the absorption peak of the C=C bond of the benzene ring, and *I*_1375_ is the height of the C–CH_3_ absorption peak.

A differential scanning calorimeter was used to measure the thermal parameters of the material. Under nitrogen gas flowing at a ratio of 20 mL·min^−1^, a sample of the material (5 mg) was then heated from 40 °C to 200 °C at a ratio of 10 °C·min^−1^, held at 200 °C for 3 min to eliminate the thermal history, and then cooled to 40 °C at a ratio of 10 °C·min^−1^. The heat absorbed was measured to extract the crystallization profile. Next, the temperature was again raised to 200 °C at a ratio of 10 °C·min^−1^ for 3 min, and the heat absorbed by the sample was measured to determine the melting profile. Each sample was tested in triplicate to ensure the high accuracy of the experimental results.

The space-charge profiles of the samples were determined using pulsed electroacoustic infrared spectroscopy, with a pulse voltage and width of 400 V and 5 ns, respectively. The test material was pressurized for 10, 300, 900, and 1800 s to analyze the space-charge distribution. The test sample specifications are 100 × 100 × 0.5 mm.

The DC voltage breakdown strength was determined using a voltage breakdown tester in accordance with the GB1408.1-2006 standard, using two-column test electrodes with diameters of 25 mm and a boosting ratio of 1 kV s^−1^. The test sample specifications were 100 × 100 × 0.1 mm. The breakdown field strength *E*_B_ was calculated using Equation (2).

*E*_B_ = *U*_B_/*d* (2)

where *E*_B_ is the breakdown field strength (kV/mm), *U*_B_ is the breakdown voltage (kV), and *d* is the thickness of the breakdown point (mm).

The dielectric properties of the samples were measured in the frequency range of 10^−1^ to 10^7^ Hz, using a Concept 80 broadband electrostatic meter at room temperature.

The volume resistivity was measured using the three-electrode method in accordance with the GB/T-1410-2006 standard, using a Keithley 6517B electrometer with an applied voltage of 500 V and a pressing time of 5 min. Each sample was measured in triplicate, and its specifications were 100 × 100 × 0.1 mm.

Trap levels of PP and the grafted materials (grafting ratio: 0.88%) were measured using the thermal shock depolarization current method. Gold electrodes were sputtered on both sides of the sample prior to taking the measurement, after which the sample was polarized under a DC electric field of 4 kV·mm^−1^ at 50 °C for 30 min and then rapidly cooled to −90 °C, where it was maintained for 3 min. The polarization voltage was subsequently removed, and the sample was heated from −90 °C to 100 °C at a heating ratio of 3 °C min^−1^ to determine the trapped space-charge distribution via the sample current measurements.

## 3. Results and Discussion

### 3.1. Infrared Spectroscopy

**Figure 3:**
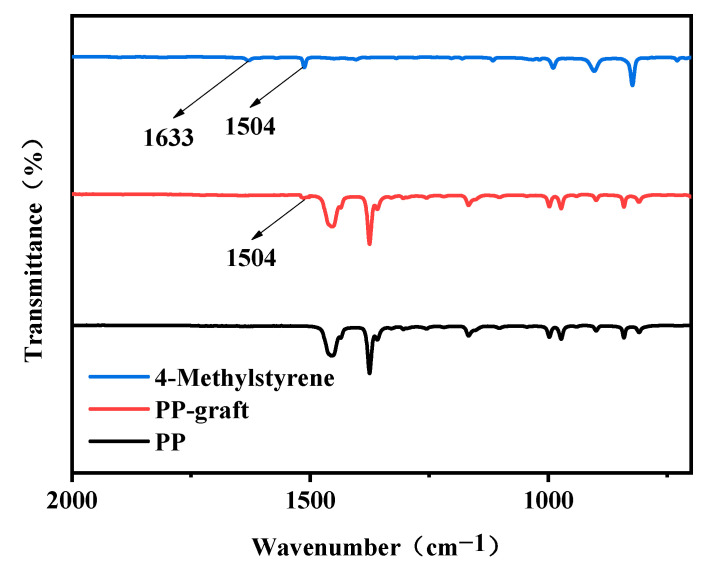
Infrared spectra of the polymer materials.

Figure 3 shows the infrared spectra of pure PP, modified PP, and 4-methylstyrene. The PP-grafted material exhibits a vibration absorption peak at 1504 cm^−1^, which is characteristic of the benzene ring in 4-methylstyrene; however, no vinyl C=C stretching vibration was observed at the characteristic absorption frequency of 1633 cm^−1^. Moreover, no new peaks corresponding to impurities were observed, confirming that the 4-methylstyrene monomer was successfully grafted onto PP.

### 3.2. DSC Analysis

**Figure 4:**
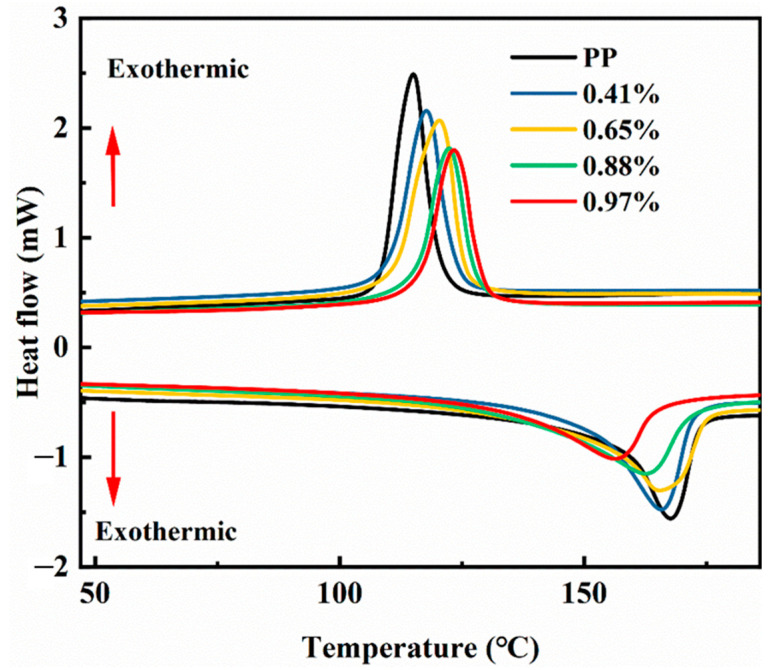
Melting and crystallization diagram.

Figure 4 shows the melting and crystallization curves of PP and modified PP with different grafting ratios. Figure 4 shows that the melting temperature of the material tends to decrease with the increasing grafting ratio; however, Figure 4 shows the crystallization temperature of the material increases with the increasing grafting ratio. As the grafting ratio increases, the structure becomes increasingly incomplete and the level of crystal imperfection becomes increasingly greater. Thus, the melting temperature tends to decrease. The increase in grafting ratio reduces the free volume inside the material, thereby increasing the steric hindrance, and the molecular motion requires more thermal energy. Thus, the crystallization temperature tends to increase. Table 1 shows that the crystallinity of the grafted product decreased from 53.2% to 39.5% as the grafting ratio increased.

### 3.3. Process Condition Analysis

**Figure 5:**
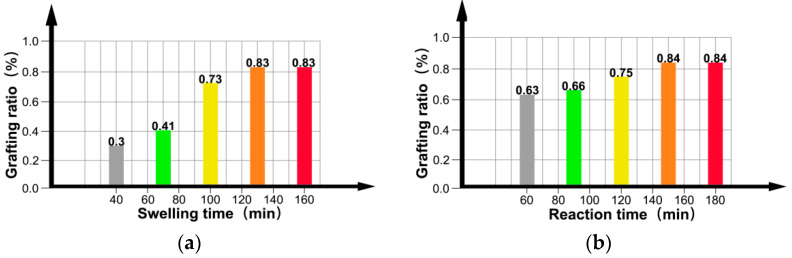

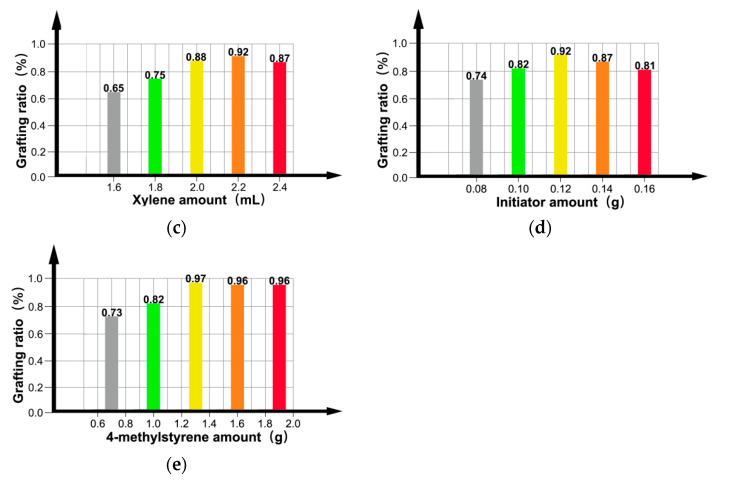
Effects of (**a**) swelling time, (**b**) reaction time, (**c**) amount of xylene, (**d**) amount of initiator, and (**e**) amount of monomer on the grafting ratio.

In the experiment where the control PP (10 g) was used with the dispersant (60 mL), Figure 5a shows the increase in grafting ratio with swelling time. Increasing the swelling time causes further wetting of xylene, which further wets and swells the amorphous region of PP, such that 4-methylstyrene is more likely to diffuse into the interior of the PP to promote the addition reaction, thereby increasing the grafting ratio. However, the area that can be wetted and swelled by xylene is limited; thus, after the optimal swelling time of 120 min, the grafting ratio levels off. Figure 5b shows that the grafting ratio increases with an increase in the reaction time. As the reaction time increased, the number of free radicals generated by the decomposition of benzoyl peroxide gradually increased. The grafting ratio initially increased gradually before saturating after 150 min, indicating that the optimal reaction time was 150 min. The diffusion of monomers and initiators into the interior of the PP is facilitated by xylene, which can wet and swell the amorphous region of PP. Accordingly, Figure 5c shows that the grafting ratio increases with the amount of xylene. However, the grafting ratio begins to decrease when the amount of xylene exceeds 2.2 mL because excess xylene dissolves some monomers and wraps PP. Thus, an excess of xylene inhibits the grafting reaction and reduces the grafting ratio. Based on our observations, the optimal amount of xylene is 2.2 mL. Figure 5d shows that the grafting ratio initially increases and then decreases with an increase in the initiator dosage because increasing the amount of initiator increases the number of decomposed free radicals, thereby increasing the grafting ratio. However, an excessive amount of the initiator causes the 4-methylstyrene to undergo a homopolymerization reaction that consumes a large amount of the monomer, and thus reduces the grafting ratio. Our observations reveal that the optimum amount of initiator benzoyl peroxide is 0.12 g. Furthermore, Figure 5e shows that the grafting ratio increases with an increase in the monomer dosage, and then remains essentially constant. Increasing the concentration of the monomer increases the probability that it will be grafted onto the main PP chain; however, this chain only has a limited number of graftable sites; thus, continuously increasing the monomer concentration beyond a certain threshold has no effect on the grafting ratio. Our results indicate that 1.3 mL is the optimal amount of monomer in this case.

In summary, to synthesize a modified PP material with an optimal graft modification at a maximum grafting ratio of 0.97%, PP (10 g), dispersant (60 mL), xylene (2.2 mL), benzoyl peroxide (0.12 g), and 4-methylstyrene (1.3 mL) are required, with a swelling and reaction time of 120 and 150 min, respectively.

### 3.4. Effect of Grafting Ratio on Volume Resistivity of the Grafted Materials

**Figure 6:**
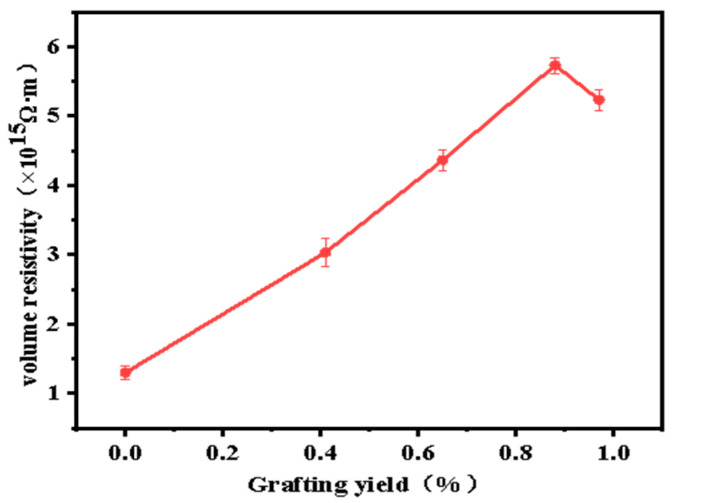
Effect of material grafting ratio on volume resistivity.

Volume resistivity is an important indicator of the insulation performance of modified PP materials. Accordingly, the volume resistivity of the prepared materials was analyzed to assess their applicability as insulating materials in HVDC cables. Figure 6 shows that the volume resistivity first increases and then decreases with the grafting ratio. A maximum volume resistivity 4.4 times that of pure PP (5.73 × 10^15^ Ω·m) was obtained at a grafting ratio of 0.88%. Continuous grafting of monomers onto PP reduces its crystallinity, but increases the amorphous fraction. However, the carriers in the amorphous area are slower than those in the crystalline area. 4-methylstyrene contains large conjugated π bonds, which increase the number of deep traps; this, in turn, hinders carrier transport in PP. Thus, the volume resistivity increased with the increasing grafting ratio; however, with a grafting ratio in excess of 0.88%, the middle of the deep traps was connected, owing to the excessive number of deep traps.

Moreover, owing to the excessive number of conjugated molecules, self-aggregation or side reactions occurred, thereby increasing the number of impurities. Thus, the carriers were influenced by the reduced barrier, resulting in a reduction in the volume resistivity.

### 3.5. Influence of Grafting Ratio on the Dielectric Permittivity and Dielectric Loss of Grafted Materials

**Figure 7:**
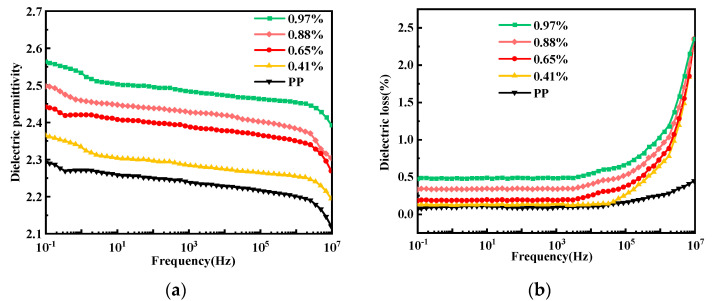
(**a**) Dielectric permittivity and (**b**) dielectric loss of the materials with different grafting ratios.

HVDC cable insulation materials require a low dielectric permittivity and low dielectric loss. Figure 7a shows that the dielectric permittivity of pure PP is approximately 2.25, while that of the grafted material increases with the increasing grafting ratio. Moreover, the number of conjugated molecules in the system increases, thereby increasing the polarity of the system and, consequently, the dielectric permittivity increases. Meanwhile, the dielectric permittivity decreases with increasing frequency because the motion of polar groups is affected by the change in electric field frequency in the high-frequency range. Figure 7b shows that the dielectric loss increases with the increasing grafting ratio, which may be due to the increase in the number of conjugated molecules and because the chain segments of the macromolecules cannot withstand the frequency of motion, resulting in the generation of macromolecules. The dielectric loss during relaxation polarization started increasing slightly at frequencies above 10^5^ Hz, possibly owing to the energy loss resulting from the increased relaxation time.

### 3.6. Effect of Grafting Ratio on Space Charge of Grafted Materials

Space charges accumulate in the grafted materials under the action of a strong electric field, resulting in a local electric field inside the material, which, in turn, leads to a break down. The current goal of research in HVDC cable insulation materials testing is to prevent the accumulation of space charges.

**Figure 8:**
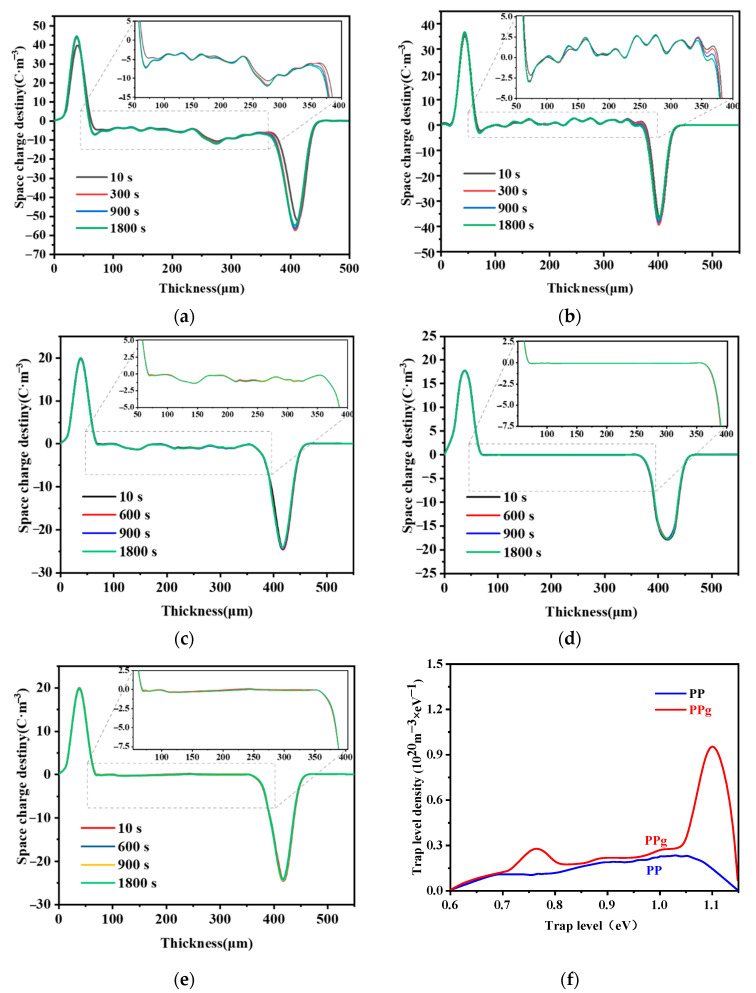
Material space charge maps: (**a**) pure PP, and grafting ratio of (**b**) 0.41%, (**c**) 0.65%, (**d**) 0.88% and (**e**) 0.97%; (**f**) material trap energy level distribution.

Under the pressurized polarization of pure PP, Figure 8a shows that the number of carriers injected into the cathode and anode increases with increasing pressurization time, which inhibits the growth and accumulation of space-charge packets and the space-charge density inside the material between 0 and 12.5 C·m^−3^. Therefore, a space-charge packet is formed inside the material under the action of a high electric field, resulting in a local effective electric field, rendering the material more prone to break down.

Figure 8b shows that grafting the 4-methylstyrene to the PP can reduce the space-charge injection at the cathode and anode. Although space-charge packets are still formed inside the material, fewer internal space-charge packets are formed than in pure PP. This indicates that the space-charge injection has been partially suppressed.

Figure 8c to Figure 8e show the internal space-charge density of the modified PP with a grafting ratio of 0.65%, which fluctuates between 0 and 1.25 C·m^−3^ and the grafting ratio of 0.88% is related to the grafting ratio. The internal space-charge density of the modified PP with a grafting ratio of 0.97% is approximately 0 C·m^−^^3^, indicating that almost no space-charge packet is formed; however, some space charge is still observed. Therefore, modified PP with a grafting ratio of 0.88% exhibits the optimal suppression of the space charges.

This observation can be explained by the introduction of the 4-methylstyrene, which reduced the crystallinity of PP and the regularity of the spherulites, blurred the boundaries, produced numerous uniformly distributed traps in both the crystalline and amorphous regions, and enhanced the charge capture ability of the deep traps, thus preventing the accumulation of space charges. Both the influence of functional groups on the crystalline and amorphous regions and the space-charge suppression ability increased with the increasing grafting ratio. However, at a grafting ratio above 0.88%, the space-charge suppression ability decreased, owing to the introduction of numerous deep traps, which not only generated channels between deep traps that facilitated the migration of opposite polar charges, but also constituted the conductivity activation energy. The ability to suppress the injection of the same polarity charge will, thus, be reduced. In summary, the ability of the material to suppress space charges first increases and then decreases with the increasing grafting ratio. Optimal space-charge suppression was achieved with a grafting ratio of 0.88%.

### 3.7. Trap Level Analysis

According to the thermal shock depolarization current measurements, Figure 8f shows that the trap energy level distributions of PP and the grafted materials both range from 0.6 to 1.1 eV, which result from the deep traps contained in the polymer materials. The maximum trap level density of the grafted material was 9.52 × 10^19^ m^−3^·eV^−^^1^, which is significantly better than that of the pure PP. Due to the incorporation of 4-methylstyrene, deep traps are introduced into the material, so the maximum trap level density of the grafted material is significantly better than that of pure PP.

### 3.8. Influence of Grafting Ratio on the Breakdown Field Strength of Grafted Materials

**Figure 9:**
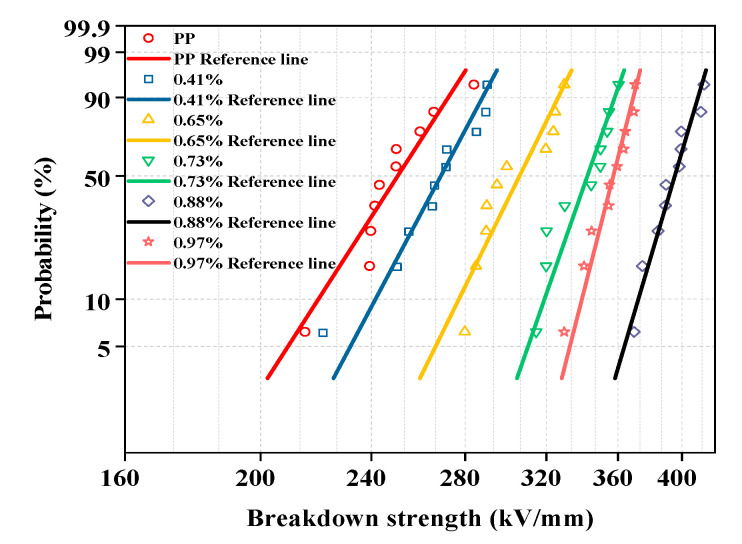
DC breakdown field strength of materials with different grafting ratios.

An insulating material can be broken down into a conductor under the action of an excessively high electric field; thus, the breakdown strength of the modified PP should be tested. Figure 9 shows a fitting diagram of the Weibull distribution of the pure and modified PP with different grafting ratios, while the characteristic breakdown values and shape factors obtained from the fit of the Weibull distribution are tabulated in Table 2. The characteristic breakdown value and shape factor first increase and then decrease. The maximum breakdown characteristic value and shape factor are both obtained at a grafting ratio of 0.88%. Table 2 shows that the breakdown field strength increased from 257.4 kV·mm^−1^ to 400.8 kV·mm^−1^ and the shape factor increased from 14.5 to 31.4. This is due to the existence of large conjugated π bonds. After 4-methylstyrene is grafted to the PP molecular chain, delocalized π electrons appeared in the system, which lead to a lower ionization potential and higher ionization potential. Molecules with high electron affinity can capture high-energy electrons, and thus prevent them from colliding and breaking molecular chains, thereby improving the breakdown field strength. However, with a grafting ratio above 0.88%, many deep traps may be formed, resulting in “tunneling” between the deep traps, accumulation of space charges, and a reduction in the breakdown field strength.

## 4. Conclusions

The aqueous suspension grafting method was used to successfully modify the insulating properties of PP. The following conditions were determined to be optimal for the process: 10 g of PP, 60 mL of dispersant, 2.2 mL of xylene, 0.12 g of benzoyl peroxide, 1.3 mL of 4-methylstyrene, a swelling time of 120 min, and a reaction time of 150 min. Modified PP with a maximum grafting ratio of 0.97% was obtained. When the graft ratio is 0.88%, it exhibited good insulating properties, the maximum volume resistivity was 4.4 times that of pure PP (5.73 × 10^15^ Ω m) and the optimal suppression of the space charges and the breakdown field strength presented a maximum value of 400.8 kV mm^−1^; however, the dielectric constant and dielectric loss were also the highest. The results indicate that the above improvements in performance can be attributed to the grafting of 4-methylstyrene and the introduction of deep traps, which significantly reduced charge mobility and the presence of delocalized π electrons in the system, which reduced the ionization potential and increased the electron affinity of the modified PP, meaning that it could trap and dissipate the energy of high-energy electrons. In summary, the modified PP is expected to provide a novel idea for the further development of HVDC cable insulation materials.

## Figures and Tables

**Table 1:** Thermogravimetric properties of the grafted materials.

Grafting Ratio (%)	Crystallization Temperature (°C)	Melting Temperature (°C)	Crystallinity (%)
0	114.5	168.1	53.2
0.41	117.6	166.0	47.3
0.65	120.6	165.1	46.8
0.88	122.2	163.7	42.1
0.97	123.5	157.1	39.5

**Table 2:** Breakdown eigenvalues and shape factors of the grafted materials with different grafting ratios.

Grafting Ratio (%)	Breakdown Field Strength (kV·mm^−1^)	Shape Factor
0	257.4	14.5
0.41	275.2	17.5
0.65	312.8	18.9
0.73	347.9	26.7
0.88	400.8	31.4
0.97	361.6	28.5

## Data Availability

Not applicable.
